# Kynurenic Acid Electrochemical Immunosensor: Blood-Based Diagnosis of Alzheimer’s Disease

**DOI:** 10.3390/bios11010020

**Published:** 2021-01-12

**Authors:** Jose Marrugo-Ramírez, Montserrat Rodríguez-Núñez, M.-Pilar Marco, Mónica Mir, Josep Samitier

**Affiliations:** 1 Department of Electronics and Biomedical engineering, University of Barcelona, Martí i Franquès 1, 08028 Barcelona, Spain; josealfonso.marrugo@icn2.cat (J.M.-R.); jsamitier@ibecbarcelona.eu (J.S.); 2 Centro de Investigación Biomédica en Red en Bioingeniería, Biomateriales y Nanomedicina (CIBER-BBN), Monforte de Lemos 3-5, Pabellón 11, 28029 Madrid, Spain; montse.rodriguez@iqac.csic.es (M.R.-N.); pilar.marco@cid.csic.es (M.-P.M.); 3 Nanobiotechnology for Diagnostics (Nb4D) Group, Institute for Advanced Chemistry of Catalonia (IQAC), Spanish National Research Council (CSIC), Jordi Girona, 18-26, 08034 Barcelona, Spain; 4 Nanobioengineering Group, Institute for Bioengineering of Catalonia (IBEC), Barcelona Institute of Science and Technology (BIST), 12 Baldiri Reixac 15-21, 08028 Barcelona, Spain

**Keywords:** Alzheimer’s disease (AD), immunosensor, kynurenic acid (KYNA), electrochemical biosensor, chronoamperometry (CA), electrochemical impedance spectroscopy (EIS), blood analysis, point of care diagnosis (PoC), in vitro diagnosis (IVD)

## Abstract

Alzheimer’s disease (AD) is a neurodegenerative disorder, characterized by a functional deterioration of the brain. Currently, there are selected biomarkers for its diagnosis in cerebrospinal fluid. However, its extraction has several disadvantages for the patient. Therefore, there is an urgent need for a detection method using sensitive and selective blood-based biomarkers. Kynurenic acid (KYNA) is a potential biomarker candidate for this purpose. The alteration of the KYNA levels in blood has been related with inflammatory processes in the brain, produced as a protective function when neurons are damaged. This paper describes a novel electrochemical immunosensor for KYNA detection, based on successive functionalization multi-electrode array. The resultant sensor was characterized by cyclic voltammetry (CV), chronoamperometry (CA), and electrochemical impedance spectroscopy (EIS). The proposed biosensor detects KYNA within a linear calibration range from 10 pM to 100 nM using CA and EIS, obtaining a limit of detection (LOD) of 16.9 pM and 37.6 pM in buffer, respectively, being the lowest reported LOD for this biomarker. Moreover, to assess our device closer to the real application, the developed immunosensor was also tested under human serum matrix, obtaining an LOD of 391.71 pM for CA and 278.8 pM for EIS with diluted serum.

## 1. Introduction

Alzheimer’s disease (AD) is the primary common cause of dementia in people over the age of 60, and the 3rd leading cause of death for the elderly population. Several AD parameters, such as incidence and prevalence, are age dependent and as the world’s life expectancy has been rising, AD has become one of the main universal healthcare problems. In 2020, more than 50 million people worldwide suffer from dementia, and is estimated to be triple in 2050 with the aging of the population [1,2].

AD is a progressive deterioration of the neuronal cells until their death, being currently incurable. This disease is mainly characterized by the abnormal accumulation of extracellular amyloid in plaques, where its main component is the amyloid beta peptide (Aβ_42_). Additionally, intraneuronal neurofibrillary tangle aggregates, formed by hyper-phosphorylated tau (P-τ) protein, are accumulated in the cerebral vasculature, with a subsequent loss of neuronal and synaptic activity in selected brain areas. Due to these alterations, astrocytes and microglia are activated, resulting in neuroinflammation and the death of neurons [3,4].

The symptoms of AD appear many years after the illness has started. A study of people with a genetic mutation that develops the disease with similar timing as the non-genetic AD patients found that the levels of Aβ_42_ were increased 22 years before symptoms, and the atrophy of their brains occurred 13 years before alarms appeared [5]. Therefore, this long asymptomatic phase causes a delay in the AD diagnosis, which happens at an advanced stage, reducing the possibility of a successful prognosis. Consequently, a key factor would be to develop early detection methods for AD.

The concentration of Aβ_42_ at the beginning of the disease is comparable to heathy controls. However, the concentration of this amyloid is reduced 10 years before symptoms appear, being confirmed as a relevant biomarker in early phases. Due to the glymphatic system, which cleanses the waste molecules inside the brain, toxic neurodegenerative proteins are also present in the blood. The access to this peripheral fluid is easier, riskless, and has a lower cost than cerebrospinal fluid (CSF) extraction by lumbar puncture. However, the Aβ_42_ in blood is 10 times less concentrated than in CSF [6]. Therefore, another important point in this disease is to find early detection biomarkers in blood at detectable levels.

Inflammatory processes in the brain greatly influence several systems underlying the disease progression. One of the main biochemical alterations occurs in the kynurenine pathway of tryptophan metabolism. This pathway generates several neuroactive compounds, including the metabolite kynurenic acid (KYNA), which is synthesized from kynurenine through kynurenine aminotransferases (KAT) I and II [7]. The potential involvement of KYNA in neurodegenerative disorders (NDDs) has been described, with potential links between KYNA levels and commonly used AD biomarkers (Aβ, total tau (T-τ), and P-τ) in CSF [8,9]. Altered levels of KYNA appear in the early stages of AD, showing the ability to considerably impair cognitive functions, including memory disruption, which could be caused by an inflammation-induced increase in KYNA production in the brain [10,11,12,13]. Additionally, some experimental studies have shown several types of cognitive deficiencies, such as working memory deficiencies, contextual learning, major depressive disorders, and bipolar disorders [14,15,16,17], in response to an increased level of KYNA in the brain. On the other hand, a clinical study has demonstrated a positive correlation between cognitive function impairments and blood-based KYNA levels in AD patients [18,19,20].

Considering that the physiological concentrations of KYNA in the brain and plasma of non-primates and primates (including humans) are usually in the nM range [21,22,23], a chronic increase in the levels of KYNA contributes by warning about the presence of impairment in cognitive functions. So, there are three important factors that makes KYNA a very relevant AD biomarker; its presence in blood, its higher concentration, compared to other AD biomarkers in blood, and the most important, its altered production at the early stage of the disease, being an efficient biomarker in peripheral fluids for early detection of NDDs [24,25,26,27].

Nowadays, there are a few systems capable of accurately detecting KYNA in blood samples. In this context, the most common methods reported for the measurement of KYNA levels in biological matrices are based on chromatographic techniques. For instance, high-performance liquid chromatography (HPLC) with electrochemical detection and capillary HPLC coupled with electrospray ionization and tandem mass spectrometry [28,29]. However, HPLC with fluorescence detection seems to be the method of choice, due to its high sensitivity for measuring KYNA levels in various biological samples [8,19,30].

Some enzyme-linked immunosorbent assay (ELISA) kits are commercially available for the quantification of this compound in biological fluids, showing sensitivities that vary between 4.7 and 48.8 nM (Cloud-Clone Corp., ref: CED18Ge; Antibodies Online, ref: ABIN2949016). Although these kits are sensitive enough, they are quite expensive, and this technology is time-consuming and requires specialized personnel and equipment.

Based on the need to go further and achieve technologies that permit AD diagnosis at a low cost, along with multi-analyte detection that could be miniaturized and automatized for point of care (PoC) use, new approaches based on the integration of biosensors in lab-on-a-chip platforms are being developed, but not yet for the proposed biomarker candidate [30].

We describe in this paper the development of a robust, reliable, and non-invasive detection platform based on an electrochemical immunosensor for the analysis of KYNA concentrations in blood, to move towards an automated platform for the non-invasive early diagnosis of AD that contains different bioreceptors for the detection of blood biomarkers in an array format. The developed KYNA sensor prototype includes two detection methods: chronoamperometry (CA) and electrochemical impedance spectroscopy (EIS) and it was tested in buffer and in serum.

## 2. Materials and Methods

### 2.1. Reagents and Instruments

Potassium hexacyanoferrate (III), potassium hexacyanoferrate (II), 3H_2_O, 11-Mercaptoundecanoic acid (MUA) (98%), 11-Mercapto-1-undecanol (MU) (97%), phosphate-buffered saline (PBS), Tween 20, N-hydroxy-succinimide (NHS), potassium nitrate (KNO_3_), kynurenic acid (KYNA), anti-rabbit immunoglobulin G horseradish peroxidase (IgG-HRP) conjugate, and 3,3′,5,5′-Tetramethylbenzidine (TMB) were purchased from Sigma-Aldrich (St. Louis, MO, USA). N-Ethyl-N-(3-dimethylaminopropyl) carbodiimide hydrochloride (EDAC) and 2-(N-morpholino) ethane sulfonic acid (MES) were purchased from Fluka (Buchs, Switzerland). Absolute ethanol, sulfuric acid (H_2_SO_4_) (95–98%), sodium hydroxide (NaOH), sodium chloride (NaCl), and hydrogen peroxide (33%) (H_2_O_2_) were purchased from Panreac (Barcelona, Spain). Human serum was purchased from Merck (Darmstadt, Germany). The immunoreagents used for KYNA detection, particularly, the polyclonal antiserum As301, used as a KYNA receptor (KYNA-Ab), and the bioconjugate derived from bovine serum albumin (BSA), As301/BSA-CHNH_2_, used as pseudo-KYNA labeled to BSA (BSA-pseudo-KYNA), were developed by the Nanobiotechnology for Diagnostics (Nb4D) group with the support of the Infraestructuras Científico-Tecnológicas Singulares (ICTS) “NANBIOSIS”, more specifically by the Custom Antibody Service (CAbS, CIBER-BBN, IQAC-CSIC).

Data acquisition and analysis from cyclic voltammetry (CV), EIS, and CA were accomplished using EC-Lab software on an SP-150 potentiostat from BioLogic SAS (Grenoble, France).

### 2.2. Multi-Electrode Platform

A matrix of 5 working electrodes (WEs) of 2 mm in diameter each was manufactured and integrated with a counter electrode (CE) (2.4 mm diameter) in the center of the matrix at the same distance from all WEs. An external reference electrode (RE) Ag/AgCl reference from BAS Inc. (West Lafayette, IN, USA) completed the electrochemical cell. The array was fabricated by photolithographic patterning of the electrodes on a low-cost and flexible cyclo-olefin polymer (COP), with a thickness of 200 nm of gold. This fabrication required two different acetate masks; the first one was necessary for the fabrication of the gold electrodes, and the second one for the fabrication of an insulating layer that covers the whole array, except for the electrodes and connecting pads, to avoid damage and unspecific adsorption effects. In order to pattern the gold electrodes with the first mask, AZ 5214 photoresist was deposited on the wafer by spin-coating at 2000 rpm, giving a film of 2.5 μm in thickness. The wafer was then cured at 95 °C for 2 min, cooled for 10 min and then exposed to UV light in the mask aligner SÜSS Microtec MJB4 (Garching, Germany). The exposure dose was 17.3 mJ/cm^2^ for 11.5 s. The wet-etching of the unprotected gold was performed with AZ 400K Developer for 2 min and the developer layer was removed with Milli-Q water.

The insulating layer was fabricated using SU-8 2002 photoresist to achieve a highly resistant sensor surface, able to bear strong acid conditions required for electrode cleaning. This layer was made by pre-curing the wafer for 1 min at 65 °C and 1 min at 95 °C with the photoresist over the array and spin-coating at 3000 rpm, giving a 2 μm thickness of the SU-8 layer. The wafer was subjected to UV exposure in the mask aligner with the same dose for 4.6 s. The time and intensity of exposure were adjusted by considering the loss of power caused by the interposing materials, in this case the acetate masks. Then, the wafer was cured at 65 °C for 1 min and at 95 °C for 2 min. The wet-etching process was performed with SU-8 Developer for 1 min and the developer layer was removed with isopropanol.

### 2.3. Immunosensor Construction

Before the functionalization of the sensor surface and the electrochemical characterizations, the electrodes were cleaned both chemically and electrochemically. For the chemical cleaning, piranha solution was used in a ratio of 1:5 of H_2_O_2_ and H_2_SO_4_. After the deactivation of the solution (20 min), the electrode region was immersed in this deactivated solution for 30–45 s. For the electrochemical cleaning, a ten-repetitive cyclic voltammogram was performed from −0.2 V to 1.6 V, using a scan rate of 150 mV/s in a solution of 0.5 M H_2_SO_4_ (see Supporting Information Figure S1).

#### 2.3.1. Fabrication of Self-Assembled Monolayer Platform for Competitive Immunosensing

The electrochemical biosensor was constructed by immersing the array in a mixed solution consisting of a 1:4 solution of 5 mM MUA and 5 mM MU in absolute ethanol for 2 h to generate a self-assembled monolayer (SAM) of these compounds on the gold electrode, followed by the respective activation of its carboxyl groups by placing 200 μL of 100 mM NHS and 300 mM EDAC in a 1:1 *v*/*v* ratio over the electrodes for 15 min. Finally, the electrode region was immersed in a stirred solution of 0.5 M MES buffer for 15 min, to clean the surface and avoid unspecific adsorption. Afterwards, 200 μL of 0.01 mg/mL of BSA-pseudo-KYNA was placed over the electrode surface for 1 h. Once the BSA-pseudo-KYNA was covalently bound to the activated carboxylic groups of the SAM, a competition assay was performed.

**Figure 1:**
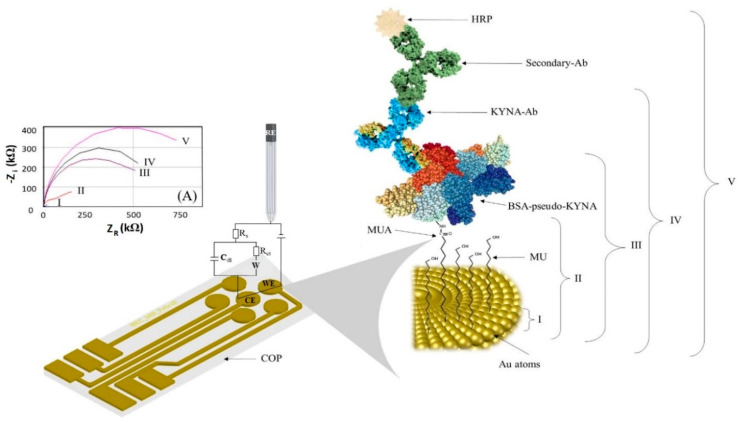
Schematic representation of the electrochemical impedance spectroscopy (EIS) electrochemical characterization of the functionalized sensor. (A) Nyquist plot for each respective immobilization (blue: bare Au, red: SAM, purple: BSA-pseudo-KYNA, black: KYNA-Ab, fuchsia: secondary-Ab).

A mixture of KYNA and the primary antibody specific to the analyte, KYNA-Ab, was incubated on the sensor for 1 h. Based on the mechanism of this type of competitive assay, at higher concentrations of KYNA, there is less interaction between the KYNA-Ab and the BSA-pseudo-KYNA conjugate attached to the electrode’s surface. Finally, an anti-IgG-HRP antibody (secondary-Ab) was added for 1 h for chronoamperometric measurements. A structural scheme of the functionalized region is shown in Figure 1. It is worth clarifying that between each step, a cleaning process was performed with Milli-Q water and 10 mM PBST, which consisted of 100 mM PBS and 5% (*v*/*v*) Tween 20.

The sensor was tested with sterile-filtered serum from plasma type AB of a human male undiluted and diluted in PBS 1/10, containing known concentrations of KYNA, to check the sensitivity of the developed sensor in more complex matrices, closer to real samples.

#### 2.3.2. Experimental Setup and Electrochemical Measurements

CV, EIS and CA were the electrochemical techniques used for the characterization of the developed sensor. For the CV measurements, a three-repetitive cyclic voltammogram was applied from −0.2 V to 0.8 V, using a scan rate of 30 mV/s. The EIS analysis was performed over a range of frequencies from 1 mHz to 200 kHz, using a modulation voltage of 10 mV versus Ag/AgCl RE. For both CV and EIS, the measurements were performed in a solution of 5 mM of K_3_[Fe(CN)_6_]/K_4_[Fe(CN)_6_] in 1 M KNO_3._ The CA was performed by applying a voltage of −0.2 V for 40 s in a TMB liquid substrate solution containing hydrogen peroxide to monitor the peroxidase reduction present as a label in the secondary antibody. For this analysis, we must consider the redox activity of KYNA, but its anodic potential at +1.26 V [31] is far from the applied voltage, not affecting the CA detection.

## 3. Results and Discussion

The KYNA biosensor was designed as a competitive indirect immunosensor, considering the small size of the analyte.

This competition assay is based on the immobilization of BSA-pseudo-KYNA on the sensor surface that competes for the interaction of KYNA-Ab with the KYNA target. A higher concentration of KYNA generates a lower amount of KYNA-Ab interacting with BSA-pseudo-KYNA on the sensor. The presence of KYNA-Ab is measured in EIS by the change in the charge-transfer resistance (R_ct_) and in the case of CA, the addition of HRP-labeled secondary antibody is required, which selectively interacts with KYNA-Ab and is detected by reduction of HRP by adding TMB and applying −0.2 V.

First, the sensor manufacturing steps were characterized to ensure correct bonding of each molecular layer on the sensor and the concentration of each of these layers was optimized to ensure efficient sensor performance. Finally, the KYNA detection performance of the sensor in buffer and in serum was analyzed.

### 3.1. Functionalization Optimization and Characterization of the Electrochemical Immunosensor

CV shows high current peaks produced in the [Fe(CN)6]^−4/−3^ (ferricyanide) redox when the electrode surface is empty, which are drastically reduced when the sensor is functionalized with the MUA/MU SAM by effective coverage of the sensor surface. However, small differences are observed in the upper layers, as it is a limited method to characterize all the manufacturing steps of the sensor (see Supporting Information, Figure S2).

EIS measures the charge-transfer resistance and double layer capacitance generated in the electrochemical cell, due to the biomolecules attached to the electrode’s surface. A redox diffusional mediator, ferricyanide, was used in solution to enhance differences between the deposited layers, which block the ferricyanide diffusion onto the surface of the electrode, generating an equivalent electrical Randles circuit (1) in the electrochemical cell.
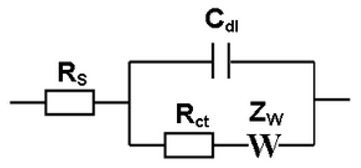
(1)
where R_s_ is the resistance that offers the electrolyte in the solution to the current flow, R_ct_ is the charge-transfer resistance produced by molecules attached on the sensor surface. C_dl_ is the double layer capacitance generated on charged surfaces like the electrode or the molecules attached on it and Z_W_ is the Warburg element, an impedance related to the diffusion process in a faradaic reaction, in this case due to the presence of ferricyanide [32]. Each layer deposited on the electrode surface is visible due to an increase in R_ct_, related to the thickness and coverage of the layer, which hinders the electron transfer from the ferricyanide. A typical Nyquist plot can be observed in Figure 1, along with the rise in the values of the R_ct_, due to the increased layers of biomolecules immobilized on the surface. Nyquist plots were fitted with the Randles model to obtain R_ct_ and C_dl_ values.

We first characterized the bare sensor, where there was negligible obstruction for the ferricyanide redox molecules to reach the electrode surface. Then, the gold was covered by the self-assemblage of thiolated linkers, through which strong molecules bring an anchoring site for the next layer and protection against nonspecific fouling of unwanted molecules.

Considering that the electron transfer reaction from ferricyanite occurs only in bare areas on the electrode surface, then the R_ct_ can be correlated to the apparent fractional coverage of the electrode surface with the next equation [33]:

(2) $$\Gamma =\frac{R_{\mathrm{ct}}^{\mathrm{SAM}}-R_{\mathrm{ct}}^{\mathrm{Au}}}{R_{\mathrm{ct}}^{\mathrm{SAM}}}\;$$

where Γ is the surface coverage, R_ct_^Au^ is the R_ct_ on the bare electrode and R_ct_^SAM^ is the R_ct_ under the same conditions measured on the MU/MUA functionalized electrode. According to the Nyquist plots results, a surface coverage value of 0.83 (*n* = 5) was calculated.

This efficient coverage of the electrodes provides a large and homogeneous surface binding site density for the BSA-pseudo-KYNA molecules.

Afterwards, the carboxylic anchoring sites from the MUA were activated as NHS esters for its subsequent interaction with the free available amino groups of the BSA-pseudo-KYNA. In this bioconjugate, the BSA protein incorporates the hapten CHNH_2_, which is a structurally related KYNA compound, allowing its recognition by the antibody produced against KYNA. The immobilization of this bioconjugate (approx. 3–5 nm in diameter) was clearly observed on the sensor surface, producing a 25-times higher ferricyanide blockage than the previous SAM layer. Next, KYNA-Ab interactions were also readily noticed by the increase in electrode impedance, as shown in Figure 1.

Optimizing the concentration of the deposited biomolecules on each functionalization step of the sensor will significantly improve its final sensitivity and selectivity to KYNA. Therefore, several concentrations of KYNA-Ab were analyzed. Since a competitive assay is required, it is worth clarifying that each characterization was performed in the absence of KYNA, to ensure BSA-pseudo-KYNA/KYNA-Ab interactions and the subsequent binding with the secondary-Ab.

#### 3.1.1. Effect of BSA-Pseudo-KYNA Concentration

The homogeneous distribution and orientation of BSA-pseudo-KYNA has a marked influence on the sensor sensitivity and selectivity. EIS can provide this important information, while CA is very relevant to determine the amount of bioreceptors attached to the sensor’s surface. Therefore, these two techniques were employed to characterize the optimum concentrations of BSA-pseudo-KYNA. On the one hand, the characterization of the sensor surface with EIS was carried out by measuring the charge transfer resistance on the surface of the functionalized sensor, which hinders the transfer of ferrocyanide electrons. On the other hand, chronoamperometry measures the current produced by the reduction of the HRP label attached to the secondary antibody.

**Figure 2:**
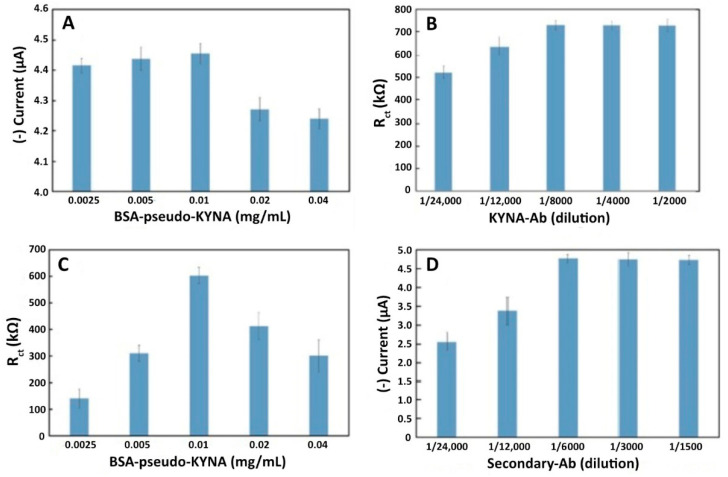
Chronoamperometry (CA) and EIS characterization of the optimization of the biosensor’s different layers. (**A**,**B**) BSA-pseudo-KYNA concentration optimization for sensor functionalization, characterized by CA and EIS, respectively; (**C**) KYNA-Ab concentration optimization using EIS in the characterization; (**D**) secondary-Ab concentration optimization characterized with CA.

The results obtained with CA are shown in Figure 2A. At first, there was an increase in the current from 4.42 to a maximum of 4.47 µA when the concentration of bioconjugate on the surface was increasing. However, concentrations higher than 0.01 mg/mL caused a decreasing signal behavior because a higher concentration of biomolecules in solution may disrupt the tidy formation of the monolayer, making it counterproductive. The results obtained with EIS, in Figure 2B, confirmed this behavior and the aforementioned Gauss distribution was even clearer, with a maximum R_ct_ of 600.02 kΩ. Therefore, the optimum concentration for this biomolecule layer was 0.01 mg/mL.

#### 3.1.2. Effect of KYNA-Ab Concentration

The KYNA-Ab plays a pivotal role on the selectivity of the biosensor because it can bind to the corresponding epitope of either the BSA-pseudo-KYNA conjugate or the KYNA analyte, which will greatly influence the resulting current signal and the ability of the sensor to detect low KYNA concentrations. As this is a competitive assay, the antibodies that bind to the BSA conjugate are the ones producing an increase in R_ct_ in EIS or an increase in current signal in CA, after the anti-IgG-HRP conjugate interacts in the system. To optimize the concentration of this antibody, the sensor response was investigated with different dilutions in 10 mM PBST.

As can be seen in Figure 2C, the higher the KYNA-Ab concentration, the higher the sensor response, down to a saturation point on dilutions over 1/8000 with a maximum current of 4.55 µA (see Supporting Information Figure S3A). This is because, at this dilution ratio, there would be no more epitopes for the binding of the antibody; thus, the unbound molecules would be washed away. The same behavior was seen in the EIS measurement, in which the R_ct_ value is kept almost constant after it achieves the saturation point on dilutions over 1/8000 with a maximum R_ct_ of 738.1 kΩ.

Based on these results, the optimum dilution ratio of the primary antibody was 1/8000. This concentration was used for further optimizations.

#### 3.1.3. Effect of Secondary Antibody Concentration

The anti-IgG-HRP conjugate is a crucial biomolecule for the biosensor response, since it introduces the enzymatic label required for the CA measurement. Five different dilutions were also tested, and the same behavior as the primary antibody was seen. As expected, the anti-IgG-HRP conjugate only binds to the KYNA-Ab, and therefore has the same tendency. As shown in Figure 2D, at dilutions lower than 1/6000, a saturation of the sensor signal was also experienced, with a maximum current of 4.61 µA obtained in CA and a maximum of 800.12 kΩ Rct in EIS (see Supporting Information Figure S3B). Both CA and EIS measurements proved this behavior. Therefore, the chosen dilution ratio for this antibody was 1/6000.

### 3.2. Study of Biosensor’s Performance

#### 3.2.1. Selectivity Study

To study the sensor signal specificity, and detect the undesired nonspecific adsorption of other molecules, several controls were performed. It is worth clarifying that every control included all the sensor steps, except the one present in the control name.

**Figure 3:**
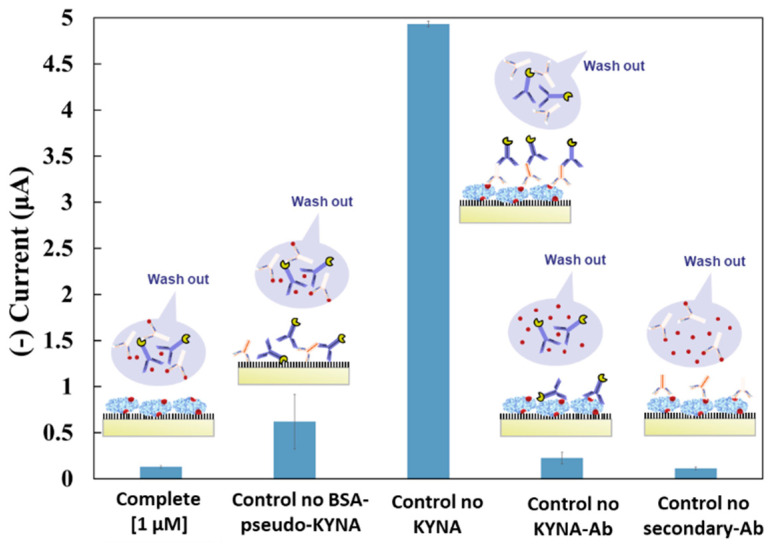
KYNA immunosensor selectivity test characterized with CA. A scheme of the results represents the possible molecules on the sensor surface and washed out after incubation.

In a competitive assay, the presence of the target (Complete, Figure 3) corresponded to the lowest signal compared with the absence of KYNA (Control no KYNA, Figure 3), where KYNA-Ab was bound to the BSA-pseudo-KYNA on the surface instead of the target, as can be appreciated by the remarkably higher signal obtained from this control.

The other controls were in a low range of current signal. The control without BSA-pseudo-KYNA (Control no BSA-pseudo-KYNA, Figure 3) provided the signal corresponding to the nonspecific absorption of KYNA-Ab and secondary-Ab on the sensor surface. This high signal was due to the excellent properties of BSA as an unfolding agent. However, this control did not affect the sensor performance, since BSA-pseudo-KYNA would be present on the surface of our sensor. Meanwhile, the control without KYNA-Ab (Control no KYNA-Ab, Figure 3) was related to a low nonspecific adsorption of anti-IgG-HRP. The last control (Control no secondary-Ab, Figure 3) was related to the background noise from the CA detection, where no anti-IgG-HRP was added, and thus no amperometric label (HRP) is present on the sensor.

#### 3.2.2. Sensitivity Study

Based on the results obtained in the optimization of the concentration of each immunoreagent, the sensitivity and linearity in the KYNA detection of the developed electrochemical immunosensor were evaluated by two different techniques, CA and EIS, using different concentrations of KYNA standard (0, 10 pM, 100 pM 1 nM, 10 nM, 100 nM, and 1000 nM) and five repetitions of each one. Each concentration of KYNA was mixed with KYNA-Ab and incubated on the sensor, where the BSA-pseudo-KYNA was immobilized. In the case of CA, incubation with labeled secondary-Ab HRP and the addition of TMB are also required. In CA, the values were normalized based on the blank value.

**Figure 4:**
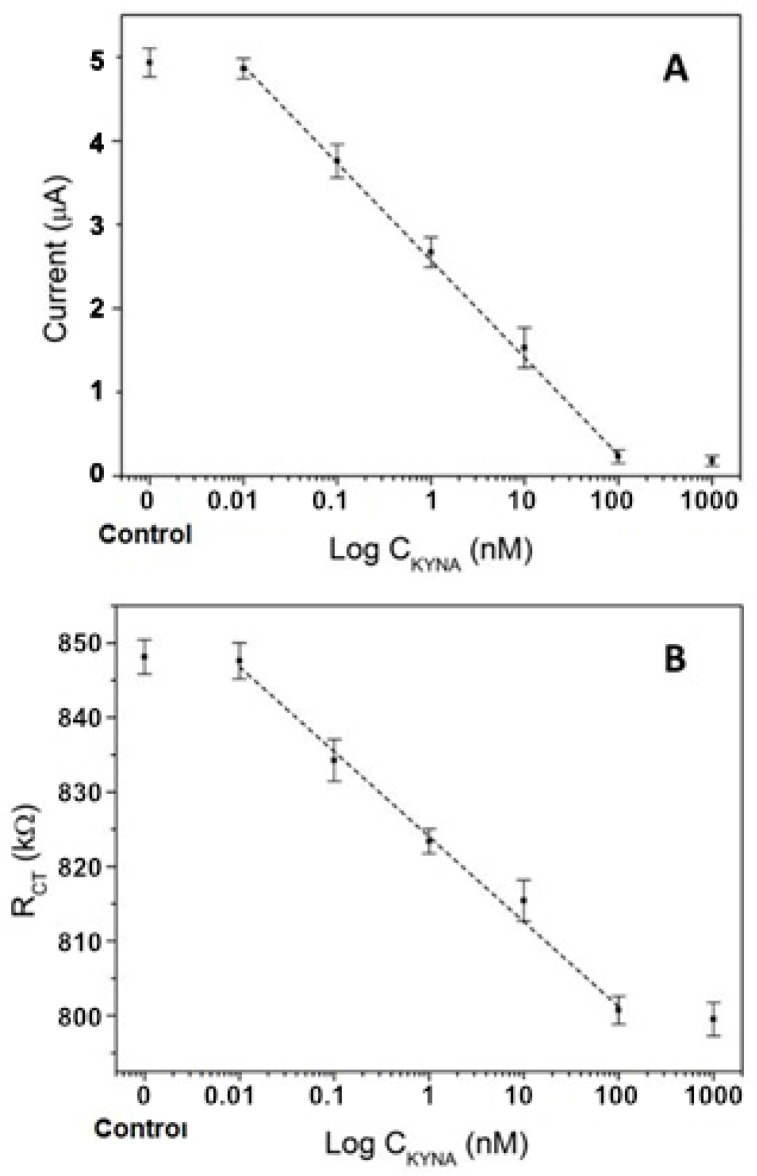
KYNA immunosensor sensitivity test in buffer. The calibration curves were performed with CA (**A**) and EIS (**B**). The concentrations considered were 0, 10 pM, 100 pM 1 nM, 10 nM, 100 nM, and 1000 nM (*n* = 5).

In CA, the current decreased as KYNA concentrations increased (Figure 4A). In a competition assay, the KYNA-Ab competes for the interaction of the analyte and the BSA-pseudo-KYNA, producing a lower amount of KYNA-Ab on the sensor surface at a higher concentration of KYNA. Therefore, a lower current is produced by a smaller amount of secondary-Ab-HRP attached to the sensor surface. The linear region was evident within the sensor working range of 0.01–100 nM with a current range of 4.93 to 0.18 µA and a calibration equation of I(µA) = 2.32 − 0.499 log C(nM) and an excellent correlation coefficient of 0.9987, with *n* = 5. The limit of detection (LOD) was calculated considering a 99.9% confidence level (k = 3) and K 10 for the limit of quantitation (LOQ) (Long; 1983), obtaining an LOD of 16.9 pM and 91 pM for the LOQ in the immunosensor KYNA detection with CA in buffer. 

Similar calculations were done for the EIS results, and the behavior observed in this technique was comparable to CA, as shown in Figure 4B. There was a decrease in R_ct_ when the concentration of KYNA increased. In this case, the R_ct_ is produced by the presence of KYNA-Ab adhered to the surface of the sensor, which hinders the electron transfer of ferricyanide. Therefore, the higher concentration of KYNA reduces the presence of KYNA-Ab on the sensor surface, resulting in a lower R_ct_.

A linear regression was done for the same range of concentrations, obtaining an R_ct_ range of 848.14 to 799.51 kΩ, with a calibration equation of R_ct_(kΩ) = 23.8 − 4.88 log C(nM) with an excellent correlation coefficient of 0.9921 (*n* = 5).

Additionally, a low LOD of 37.6 pM and an LOQ of 1.22 nM were obtained. According to the values obtained for the LOD and LOQ in both techniques, it can be concluded that the CA can provide a better sensing of KYNA under buffered conditions. However, the main drawback of this technique, compared to the EIS, is that it requires an extra immobilization step of the anti-IgG-HRP conjugate after the KYNA-Ab. Therefore, CA is more time-consuming and less cost-effective than EIS, and requires extra reagents, i.e., anti-IgG-HRP and TMB, to label the analyte interaction.

EIS can be more easily adapted to a lab-on-a-chip device since it is label free and needs fewer steps and reagents for sensing. Therefore, the EIS technique could be considered as a better sensing technique with an excellent LOD for KYNA in buffer conditions. This sensor offers an excellent platform to construct a low-cost and easy-to-use array, able to perform diagnosis based on KYNA detection.

#### 3.2.3. Determination of KYNA in Human Serum Samples

With the aim to test the sensitivity of the developed sensor in more complex matrices, closer to real samples, the measurements were also performed in undiluted human serum. The same range of KYNA concentrations was used. A summarizing table with the KYNA LOD and LOQ in buffer, serum, and 1/10 serum dilution measured by CA and EIS is presented in the Supporting Information (Table S1).

**Figure 5:**
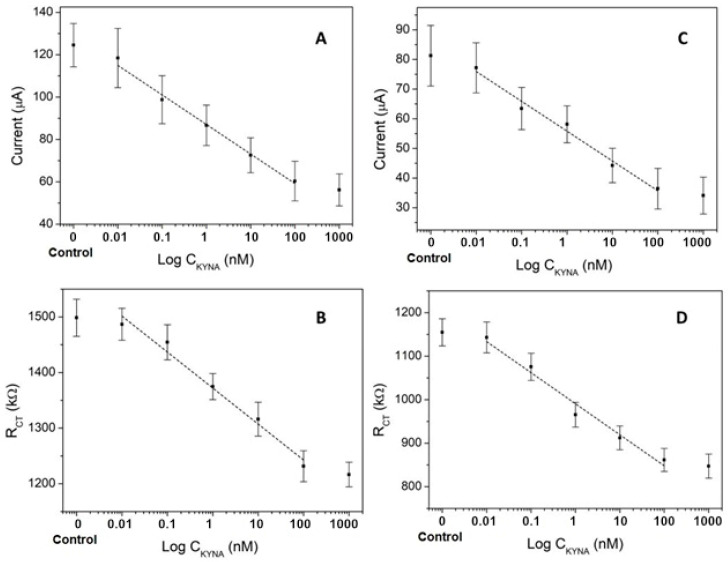
KYNA immunosensor sensitivity test. (**A**,**B**) Calibration curves in undiluted human serum, using CA and EIS, respectively, and (**C**,**D**) calibration curves in 1/10 diluted human serum in phosphate-buffered saline (PBS), using CA and EIS, respectively. The concentrations considered were 0, 10 pM, 100 pM 1 nM, 10 nM, 100 nM, and 1000 nM (*n* = 5).

A clear difference observed from the results with serum (Figure 5) compared with buffer KYNA detection is the higher dispersion of the results, with the immediate outcome of a standard deviation increment of about 10 times in serum. Considering the presence of multiple biomolecules in the serum samples, these molecules are a new variable affecting on the sensor response. Consequently, the LOD in CA and EIS detection is also impaired. As can be seen in Figure 5A, the LOD obtained in serum for CA was approx. 50 times higher than the LOD in buffer; 818.1 pM vs. 16.9 pM, respectively. An evident linear region within the same working range (0.01–100 nM) was observed, with a calibration equation of I(µA) = 37.172 − 6.1874 log C(nM), with an excellent correlation coefficient of 0.9913 (*n* = 5).

This increment in the LOD could be due to the complex nature of the matrix, which contains a high level of interfering compounds that interact with the antibody receptor [34] and the nonspecific adsorption of matrix proteins that cover the BSA-pseudo-KYNA, reducing the binding options for KYNA-Ab and anti-IgG-HRP, with the consequent decrease in the sensor sensitivity [35,36].

As observed in the CA measurement, there was also a remarkable increase in the LOD of EIS in serum (365 pM) (Figure 5B), which it was almost 10 times more than in buffer (37 pM). The evident linear region within the same working range has a calibration equation of R_ct_(kΩ) = 125.81 − 28.168 log C(nM), with a good correlation coefficient of 0.9829 (*n* = 5).

The greater effect of the matrix on CA sensitivity than in EIS may be due to passivation with nonspecific adsorbed molecules that block the electron transfer from the redox label to the electrode and the presence of redox active molecules in the serum matrix that interfere in HRP detection.

This increase in R_ct_ is mainly due to the nonspecific adsorption of some component of the serum on the surface of the electrode. The thickness of this layer of undesired adsorbed molecules increases the current resistance in the electrode. Additionally, this new layer blocks the BSA-pseudo-KYNA interaction points with the KYNA-Ab. Thus, the KYNA-Ab binding is lower and the recorded R_ct_ has a higher background signal from the nonspecific adsorption of serum molecules, making it less detectable and reducing the sensitivity of the sensor. However, the nonspecific layer formed by the serum has much less impact in R_ct_ recorded with EIS than the one in CA.

As can be seen in Figure 5C,D, when the undesired layer of molecules from the serum is reduced, diluting this sample 1/10, there is a significant decrease in the matrix effect that is causing the serum insulation on the sensor, obtaining a much better LOD from both techniques; 391.71 pM for CA and 278.8 pM for EIS.

For the CA measurement, a calibration equation of I(µA) = 25.369 − 4.374 log C(nM), with an excellent correlation coefficient of 0.9848 (*n* = 5), and in the case of EIS, a calibration equation of R_ct_(kΩ) = 163.23 − 31.557 log C(nM), with a correlation coefficient of 0.9791 (*n* = 5), were obtained.

Based on these results, it was necessary to evaluate the nonspecific serum interferences occurring on the electrode. For this purpose, the sensor platform modified with SAM and BSA-pseudo-KYNA was put into contact with the serum (without the antibody addition step), to determine the grade of protein adsorption on the surface.

This approach was done using the two techniques, and the results are shown in Figure S4 (see Supporting Information). In these results, the remarkably high resistance that was generated by the adsorption of serum proteins is evident, which reduced the analyte interaction properties of the sensor. This fact was even more appreciated with an incubation with the secondary-Ab, and its nonspecific adsorption followed the same trend as the serum adsorption, as shown in the CA detection. As expected, this behavior diminished when a 1/10 serum dilution was done. This dilution of the KYNA concentration was already considered for the calculation of the LODs.

Even with this increase in the LODs observed in serum, this sensor platform has an excellent sensitivity and working range, for the appropriate detection of KYNA in blood for AD diagnosis.

Based on the studies reported in the literature about the detection of this specific biomarker in a blood-based scenario, it is valid to state that the electrochemical immunosensor developed in this study presents an excellent LOD. As mentioned in the introduction, HPLC is the current gold standard technique to determine KYNA with different detectors, such as fluorometric and electrochemical. By using an HPLC coupled with a fluorescence detector, the concentrations obtained for KYNA in human serum for a control group and AD patients were 30.94 ± 13.18 nM and 20.85 ± 8.23 nM, respectively [20]. Commercially available ELISA kits have also been used for this purpose, giving LODs varying between 4.7 and 48.8 nM (Cloud-Clone Corp., ref: CED18Ge; Antibodies Online, ref: ABIN2949016). A recent study reported an optimal cut-off point for the determination of KYNA of 39.96 nM in CSF [9]. In plasma, KYNA was also considered a significant predictor of AD. A preclinical study of patients with AD demonstrated a higher content of KYNA (above 50 nM) compared to healthy participants [37].

The developed electrochemical immunosensor has demonstrated a lower LOD, of 278.8 pM, with EIS in diluted serum samples than that reported in the literature for KYNA detection with serum samples, which is about 17 times lower than the lowest LOD reported. Moreover, these outstanding results have been achieved with a label-free configuration, that requires fewer steps and reagents and it is performed with EIS electrochemical detection, which makes this technology easy to miniaturize, automatize, and integrate in a multiplexed platform at a low cost, which are features required for PoC development.

This work takes us a step closer to a multiple array analysis integrated for PoC diagnosis of AD in blood. This low-cost technology brings the possibility of a massive test of AD before symptoms appear to achieve an early detection of this disease, which is critical to achieve a high likelihood of AD cure.

#### 3.2.4. Reproducibility Analysis of the Sensor

We obtained good reproducibility, with is low standard deviation with five repetitions, when the sensor was tested under controlled conditions of standard buffer solution; 4.86 ± 0.12 µA (for 0.01 mM of KYNA measured with CA in buffer), which is 2.46% of the signal measured. However, when the sensor was tested in serum samples, a more complex matrix came into play, resulting in an increase in the standard deviation of the measurement in both detection methods; 77.18 ± 10.9 µA (for 0.01 mM of KYNA measured with CA in 1/10 serum), which is 10.95% of the signal measured, and 118.45 ± 14.0 µA (for 0.01 mM of KYNA measured with CA in serum), which is 11.82% of the signal measured.

The lower sensor signal (higher KYNA concentration) also increases the standard deviation. For example, in CA detection in 1/10 serum, a lower signal was obtained with 1000 nM; 34.10 ± 6.21 µA has a higher standard deviation (18.21%) than that obtained with 0.01 nM; 77.18 ± 8.45 µA (10.95%). The same tendency is observed in the different sample matrices tested and detection methods used.

Moreover, it is important to point out the large differences in reproducibility observed with EIS compared with CA, using the same sensor in all the sample matrices tested, but with different methods of detection. Comparing the 0.01 mM analysis in buffer of KYNA, EIS is almost 10 times more reproducible than CA; 847.59 ± 2.43 kΩ (0.28%) and 4.86 ± 0.12 µA (2.47%), respectively. Reproducibility is another important feature that makes EIS a better candidate for KYNA detection.

## 4. Conclusions

This work has demonstrated an effective immunosensor platform for the detection in buffer and human serum samples of KYNA, which is considered a blood-based biomarker candidate for AD.

The EIS characterization of the sensor fabrication steps demonstrated an optimal surface coverage of 0.83 for the MU/MUA SAM and a good immobilization of all sensor layers.

The results obtained suggest a highly reproducible detection by EIS with 0.28% of the signal, an increased value in CA measurements (2.47%) and in samples with serum matrix (11.82%, for the same KYNA concentration in CA).

A good sensitivity and a high molecular specificity were successfully reached by optimizing each biosensor layer concentration. This platform has demonstrated an excellent LOD; 16.9 pM and 37.6 pM for CA and EIS, respectively, in buffer, which is the lowest LOD reported in the literature for KYNA detection. The measurements done in human serum confirm the performance of the sensor when complex matrices are used. The nonspecific matrix interference was minimized by a 1/10 serum dilution, obtaining an LOD of 391.7 pM and 278.8 pM for CA and EIS, respectively.

With such results, it was possible to demonstrate good reproducibility and selectivity, as well as excellent sensitivity of the developed biosensor, in an appropriate working range for KYNA detection correlated with AD diagnosis.

Considering that CA is less reproducible and more time- and reagent-consuming, it is less cost-effective than the EIS technique. Based on the advantages EIS has over CA, EIS has been chosen as the recommended technique for this particular immunosensor application.

## Data Availability

Not applicable.

## Supplementary Materials

The following are available online at https://www.mdpi.com/2079-6374/11/1/20/s1, Figure S1: Electrochemical cleaning cyclic voltammogram, Figure S2: Characterisation of the layer-by-layer modified sensor by cyclic voltammetry, Figure S3: KYNA-Ab concentration optimization and Secondary-Ab concentration optimization, Figure S4: Matrix effect study, Table S1: Comparative table of LOD and LOQ of the CA and EIS based on several media.
