# Supplementary material for: Kynurenic Acid Electrochemical Immunosensor: Blood-Based Diagnosis of Alzheimer’s Disease
Source: Biosensors (Basel). 2021 Jan 12;11(1):20. doi: 10.3390/bios11010020 (PMC7827041; doi:10.3390/bios11010020)
Supplement: Supplementary file 1 [file biosensors-11-00020-s001.pdf]

biosensors

MDPI

SUPPORTING INFORMATION

Kynurenic Acid Electrochemical Immunosensor: Blood-based Diagnosis of Alzheimer's Disease

Jose Marrugo-Ramírez [1], Montserrat Rodríguez-Núñez [2,3], M.-Pilar Marco [2,3], Mónica Mir [1,2,4,*,†] and Josep Samitier [1,2,3,4,†]

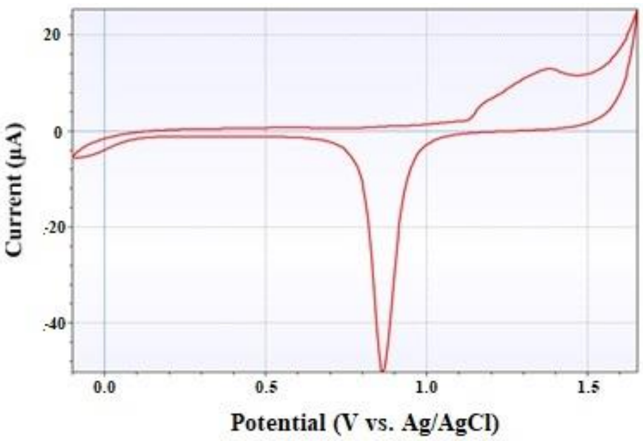

Figure S1. Electrochemical cleaning of the gold electrodes by cyclic voltammogram from -0.2 V to 1.6 V, using a scan rate of 150 mV/s in a solution of 0.5 M H_2SO_4.

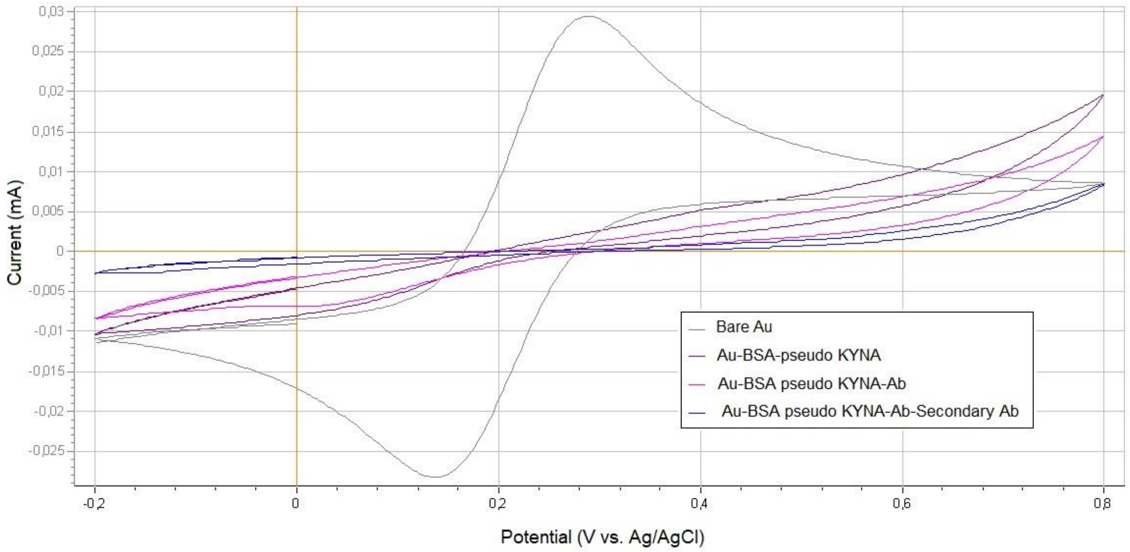

Figure S2. Characterisation of the layer-by-layer modified sensor by cyclic voltammetry in 5mM of $K_3[Fe(CN)_6]/K_4[Fe(CN)_6]$.

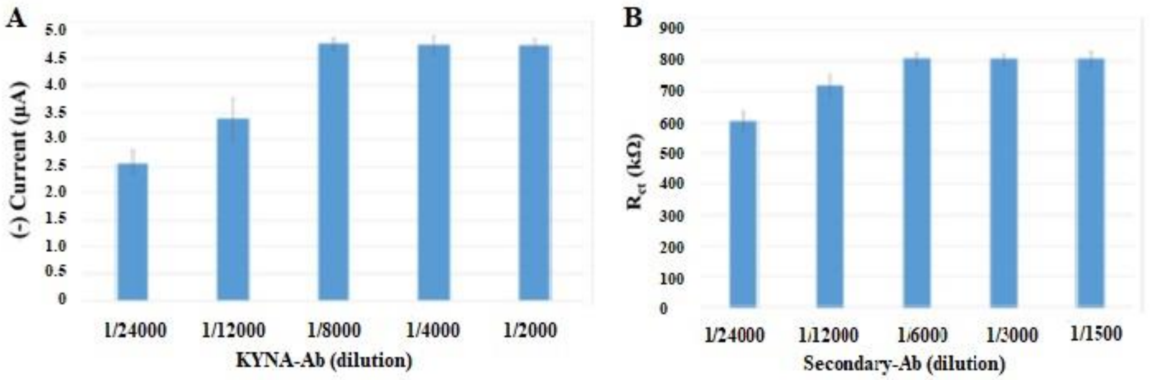

Figure S3. (A) KYNA-Ab concentration optimization for sensor functionalization, characterized by CA; (B) Secondary-Ab concentration optimization characterized with EIS.

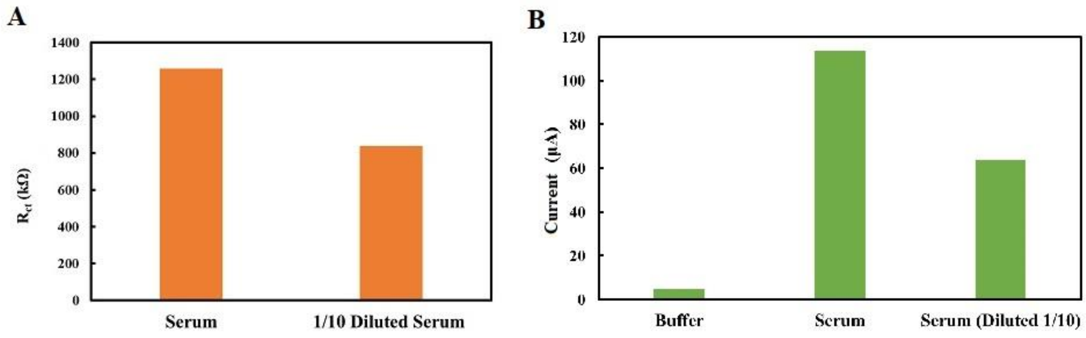

Figure S4. Matrix effect study. (A) Serum matrix adsorption on the biosensor platform (Au-SAM-BSA). (B) non-specific Secondary-Ab adsorption on different sample matrix media.

Table S1. Comparative table of LOD and LOQ of the CA and EIS based on several media.

	**Buffer**		**Serum**		**Serum (1/10)**	
	CA	EIS	CA	EIS	CA	EIS
LOD (nM)	0.016	0.037	0.81	0.36	0.39	0.24
LOQ (nM)	0.091	1.22	$6.19\cdot10^{6}$	441.6	$2.94\cdot10^{5}$	618.7
